# Deep-sea corals near cold seeps associate with sulfur-oxidizing chemoautotrophs in the family Ca. Thioglobaceae

**DOI:** 10.1186/s40168-025-02254-z

**Published:** 2025-11-13

**Authors:** Samuel A. Vohsen, Harald R. Gruber-Vodicka, Eslam O. Osman, Matthew A. Saxton, Samantha B. Joye, Nicole Dubilier, Charles R. Fisher, Iliana B. Baums

**Affiliations:** 1 Biology Department, The Pennsylvania State University, University Park, PA 16802 USA; 2 Symbiosis Department, Max Planck Institute for Marine Microbiology, Bremen, Germany; 3 Zoological Institute, Christian-Albrecht University of Kiel, Kiel, Schleswig-Holstein Germany; 4 Marine Science Program, Division of Biological and Environmental Science and Engineering, King Abdullah University of Science and Technology (KAUST), Thuwal, Saudi Arabia; 5 Department of Marine Sciences, University of Georgia, Athens, GA 30602 USA; 6 Helmholtz-Institute for Functional Marine Biodiversity at the University of Oldenburg (HIFMB), Im Technologiepark 5, Oldenburg, 26129 Germany; 7 Alfred Wegener Institute, Helmholtz-Centre for Polar and Marine Research (AWI), Am Handelshafen 12, Bremerhaven, 27570 Germany; 8 Institute for Chemistry and Biology of the Marine Environment (ICBM), School of Mathematics and Science, Carl Von Ossietzky Universität Oldenburg, Ammerländer Heerstraße 114-118, Oldenburg, 26129 Germany

**Keywords:** SUP05, Thioglobaceae, Deep-sea, Cold seep, Deep-sea corals, Chemoautotrophy, Symbiosis, Sulfur oxidation, Carbon fixation

## Abstract

**Background:**

Corals are known for their symbiotic relationships, yet there is limited evidence of chemoautotrophic associations. This is despite some corals occurring near cold seeps where chemosymbiotic fauna abound including mussels that host sulfur-oxidizing chemoautotrophs from the SUP05 cluster (family Ca. Thioglobaceae). We investigated whether corals near cold seeps associate with related bacteria and report here that these associations are widespread.

**Results:**

We screened corals, water, and sediment for Thioglobaceae using 16S metabarcoding and found ASVs associated with corals at high relative abundance (10 – 91%). These ASVs were specific to coral hosts, absent in water samples, and rare or absent in sediment samples. Using metagenomics and transcriptomics, we assembled the genome of one phylotype associated with *Paramuricea* sp. B3 (ASV 4) which contained the genetic potential to oxidize sulfur and fix carbon, and confirmed that these pathways were transcriptionally active. Furthermore, its relative abundance was negatively correlated with the stable isotopic composition of its host coral’s tissue suggesting some contribution of chemoautotrophy to the coral holobiont.

**Conclusions:**

We propose that some lineages of Thioglobaceae may facultatively supplement the diet of their host corals through chemoautotrophy at seeps or may provide essential amino acids or vitamins. This is the first documented association between chemoautotrophic symbionts and corals at seeps and suggests that the footprint of chemosynthetic environments is wider than currently understood.

**Supplementary Information:**

The online version contains supplementary material available at 10.1186/s40168-025-02254-z.

## Background

Deep-sea corals are important foundation species that support a diverse community of animals that rivals the diversity of shallow, tropical reefs and includes many commercially important fish species [1–5]. Deep-sea coral communities can be found along all continental margins from the Arctic to the Antarctic and on seamounts worldwide [6, 7]. In the deep Gulf of Mexico and in other locations, they overlap with cold seeps characterized by elevated concentrations of hydrogen sulfide and/or hydrocarbons [4, 8–13].

At cold seeps, some animals associate with chemotrophic symbionts which provide them with nutrition from the oxidation of these reduced chemical species [14–16]. Bathymodiolin mussels and vestimentiferan tubeworms obtain the bulk of their nutrition from these symbionts and form dense assemblages which in turn support highly productive animal communities [17–19]. The symbionts of mussels belong to the widespread SUP05 cluster which includes sulfur-oxidizing symbionts associated with various faunal hosts from reducing habitats such as cold seeps, hydrothermal vents, and organic falls [20–23]. Their hosts span many invertebrate phyla and include vesicomyid clams [24], scallops [25], snails [26], several groups of sponges [27, 28], terebellid polychaetes [20], and anemones [29]. The SUP05 cluster, along with the Arctic96-BD19 clade, comprise the family Ca. Thioglobaceae (Class Gammaproteobacteria) which also includes many free-living species that are abundant at oxygen minimum zones and hydrothermal vents where they dominate dark carbon fixation [23, 30–34]. Whether corals form associations with similar bacteria at chemosynthetic habitats has been a focus of research for several decades.

Early work in the Gulf of Mexico explored the trophic dynamics of fauna at cold seeps using stable isotopic compositions and found that some anemones and stoloniferous corals had tissues with low δ^13^C and δ^15^N values suggesting they receive significant nutrition from chemotrophic sources [35]. Further work with the deep-sea, scleractinian coral, *Desmophyllum pertusum* (formerly *Lophelia pertusa* [36])*,* found that they formed mounds near cold seeps where methane concentrations in the sediment were elevated. Thus, it was initially hypothesized that corals host chemotrophic symbionts or otherwise incorporate chemosynthetic primary productivity [37, 38]. Indeed, a relative of Thioglobaceae was detected in *D. pertusum* from Norway and the Gulf of Mexico using 16S metabarcoding [39, 40] and incubation experiments with *D. pertusum* detected carbon fixation [41]. However, these bacteria could not be located with FISH microscopy and showed no evidence that they were abundant in corals [42]. Other work analyzing available particulate matter and bulk stable isotopic compositions of coral tissues demonstrated that *D. pertusum* does not receive detectable nutritional input from chemosynthetic sources. It concluded that corals simply use authigenic carbonates produced at cold seeps as a substrate after seepage has waned [9, 43]. These results and the oxygen demand of chemoautotrophic symbionts have led some to suggest that cnidarians would not form associations with sulfide-oxidizing symbionts since they have no specialized respiratory structures or oxygen transport mechanisms [44]. This was recently refuted, however, by the confirmation of sulfur-oxidizing symbionts in an anemone at hydrothermal vents [29]. Further, members of the family Thioglobaceae were recently detected in mesophotic and deep-sea octocorals from the mediterranean and Gulf of Mexico highlighting the possibility of this association in octocorals at cold seeps [45–48].

Here we report the discovery of widespread associations between deep-sea corals and members of the family Thioglobaceae. We used 16S metabarcoding to screen corals, water, and sediment for the presence of Thioglobaceae. We screened 421 colonies from 42 coral morphotaxa including scleractinians (stony corals), octocorals, antipatharians (black corals), and zoanthids from deep-sea sites and included mesophotic and shallow-water coral species for comparison. We then focused on one species, *Paramuricea* sp. B3 [12], from a cold seep site within BOEM lease block AT357, as a model to study the role of Thioglobaceae in corals. We sequenced metagenomes and metatranscriptomes of *Paramuricea* sp. B3 to assemble the genome of its Thioglobaceae associate, to confirm if its genome encoded the pathways necessary for chemoautotrophy and that those pathways were transcriptionally active. Finally, we investigated the nutritional relationship between *Paramuricea* sp. B3 and its associate using carbon and nitrogen stable isotopic analysis.

## Methods

### Collections

**Fig. 1:**
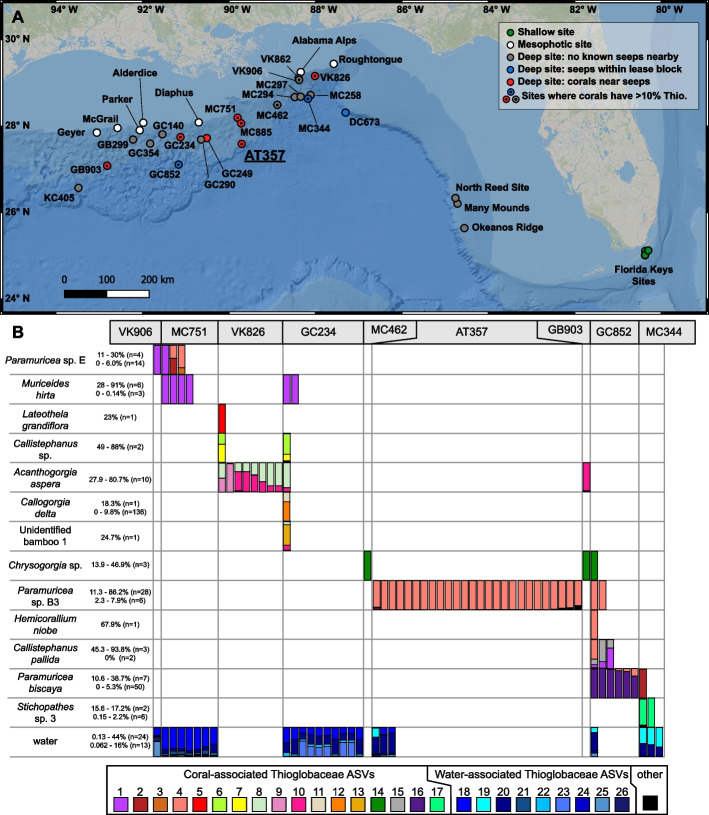
Map of sampling locations and occurrence of abundant Thioglobaceae in corals and seawater. **A** Map of sampling locations excluding sites in Curaçao. Shallow (< 20 m) and mesophotic (50–100 m) sites are displayed with green and white points, respectively. Deep-sea sites (> 200 m) are displayed gray points if no known seeps are nearby, blue points if seeps are known within the lease block, and red points if corals were sampled near signs of active seepage. Sites with corals that harbored more than 10% Thioglobaceae are denoted with an inner black point. **B** Occurrence of Thioglobaceae ASVs in coral colonies with higher than 10% Thioglobaceae as well as the Thioglobaceae ASVs in the water from those sites. Each bar represents the composition of Thioglobaceae ASVs in an individual coral or water sample. Thioglobaceae ASVs that comprised more than 10% in any coral sample or occurred in water are displayed with unique colors whereas all other Thioglobaceae ASVs are displayed in black. Samples from the same morphotaxa are organized in rows and those from the same site are organized into columns. The range of total relative abundances of Thioglobaceae ASVs for the colonies shown in the figure is reported beside each morphotaxa name as well as the range for colonies below 10%

Four hundred and twenty-one coral colonies belonging to forty-two morphotaxa were collected from thirty-one deep-sea and mesophotic sites in the Gulf of Mexico during eight cruises from 2009 to 2017 as well as shallow-water sites in the Florida Keys and Curaçao (Fig. 1A, Tables S1,S2 in Additional File 1) as described by Vohsen et al. [49]. Signs of seepage were only observed at collection sites deeper than 400 m and not at shallower sites. Signs of active seepage were spatially and temporally heterogeneous and included bacterial mats, bubbles releasing from the seafloor, and the presence of living chemosymbiotic fauna including bathymodiolin mussels, vesicomyid clams, and aggregations of *Lamellibrachia* tubeworms. However, the presence of tubeworms alone is not considered a sign of active seepage since they can persist after very little sulfide remains in the surrounding water [50, 51].

Deep-sea corals were sampled using specially designed coral cutters mounted on the manipulator arm of remotely operated vehicles (ROVs). Coral fragments were removed, placed in temperature insulated containers until recovery of the ROV, and were maintained at 4 °C for up to 4 h until preservation in ethanol or frozen in liquid nitrogen. Corals were also sampled from four shallow-water sites in the Florida Keys and one site in Curaçao from 1–20 m depth. Coral fragments were removed using either a hammer and chisel or bone cutters and placed in separate sealed plastic bags. These samples were preserved in ethanol or frozen in Liquid nitrogen on the boat or on shore within 8 h.

Sediment samples were taken using an ROV in close proximity to many of the deep-sea coral collections with 6.3 cm diameter push cores. Upon recovery of the ROV, 1 mL of sediment from the top 1 cm of each sediment core was frozen in Liquid nitrogen. In 2015, water was sampled using a Large Volume Water Transfer System (McLane Laboratories Inc., Falmouth, MA) which filtered 330–360 L of seawater through a 0.22 micron porosity filter (142 mm diameter, Table S3). One quarter of each filter was preserved in ethanol and another quarter was frozen in Liquid nitrogen for these analyses. In 2016 and 2017, 2.5 L niskin bottles were fired above corals and upon recovery were filtered through 0.22 micron Sterivex filters (MilliporeSigma, Burlington, MA) which were frozen in liquid nitrogen.

### Description of study site: AT357

**Fig. 2:**
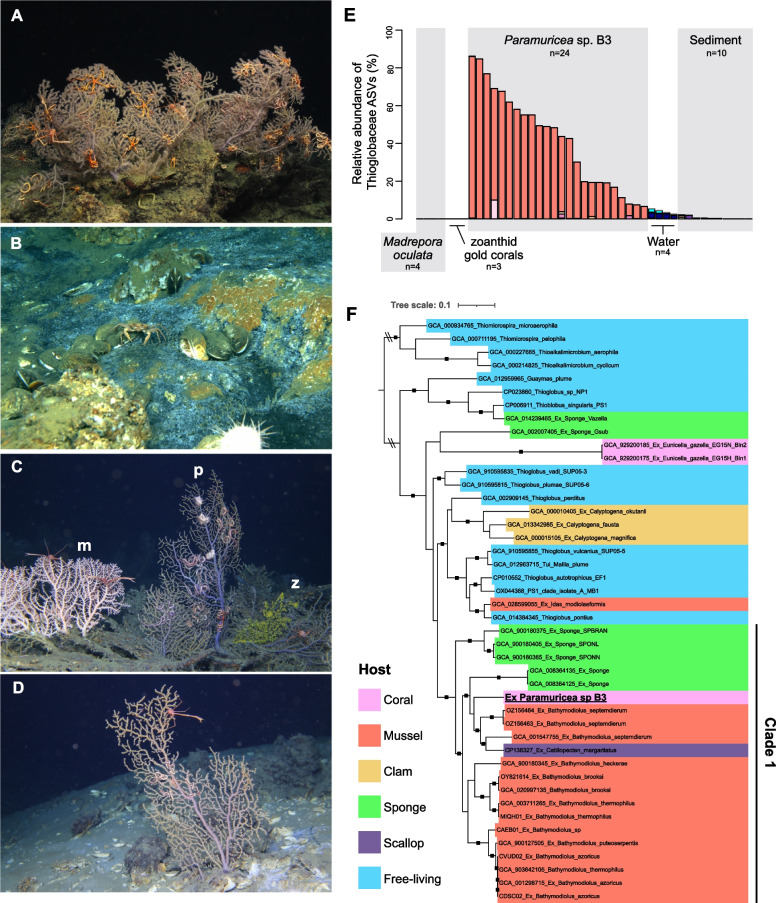
Study site AT357: contextual images, Thioglobaceae ASVs, and phylogenomic tree of Thioglobaceae. Images from AT357 of *Paramuricea* sp. B3 and associated fauna (**A**), bacterial mats and living chemosymbiotic mussels (*Bathymodiolus brooksi*) indicating active seepage of reduced sulfur species (**B**), *Madrepora oculata* (m) and zoanthid gold corals (z) co-occurring with *Paramuricea* sp. B3 (p, C), and colonies of *Paramuricea* sp. B3 growing very near bacterial mats (**D**). **E** The occurrence of Thioglobaceae ASVs in corals, water, and sediment at site AT357. The relative abundances of only Thioglobaceae ASVs are shown. Each color represents a different ASV. **F** Phylogenomic tree of Thioglobaceae genomes including the associate of *Paramuricea* sp. B3 (bold, underlined) from a concatenated alignment of 120 amino acid sequences. Nodes with greater than 95% ultrafast bootstrap support are denoted with black squares. Colors signify host type

A large community of corals, dominated by *Paramuricea* sp. B3 [12] and *Madrepora oculata* was present in BOEM lease block AT357 (27.587 N, −89.705 W) between 1045–1064 m depth (Fig. 2A-D). Some *Paramuricea* colonies were encrusted by a zoanthid gold coral (Fig. 2C). Corals from this site were used to investigate potential nutritional interactions between corals and their Thioglobaceae associates. Corals were found over an area of at least 250 by 250 m at this site and there are numerous areas of active seepage interspersed among the coral colonies (Fig. 2B,D). Colonies of *Paramuricea* sp. B3 were found on mounds of *Madrepora oculata,* and on authigenic carbonates formed in areas of previous or active seepage. Large bacterial mats were often present in small troughs and depressions between the corals. Chemosymbiotic *Bathymodiolus brooksi* mussels and vesicomyid clams were occasionally present in these areas of active seepage as well (Fig. 2B). Twenty-two *Paramuricea* sp. B3 colonies were sampled across this site including colonies growing near active seeps and others from areas with no visible indications of currently active seepage within 10 m.

### 16S rRNA gene metabarcoding

Corals, water, and sediment were screened for Thioglobaceae using 16S metabarcoding. DNA was extracted from coral tissue and sediment samples using DNeasy PowerSoil kits (Qiagen, Hilden, Germany) following manufacturers protocols using approximately 1 cm of coral branches and about 0.25 g of sediment. Exceptions include DNeasy Blood and Tissue kits applied to *Acropora palmata* and DNeasy DNA/RNA allprep kits applied to *Callogorgia americana* as well as five technical replicates each of *Callogorgia delta, Swiftia exserta, Muricea pendula,* and *Paramuricea biscaya.* DNeasy PowerSoil kits were also used for water samples collected with the McLane pump in 2015 using 1 cm^2^ of filter. Replicate extractions were performed on quarters of the filter preserved in both ethanol and frozen in Liquid nitrogen for all water samples in 2015. DNA was extracted from the Sterivex filters using Qiagen DNeasy PowerWater kits following manufacturer’s protocols. Four extraction blanks were processed using the DNeasy Powersoil kits but without any coral tissue, sediment, or water filter.

The V1 and V2 region of the 16S rRNA gene were amplified as described in Vohsen et al. [49] using universal bacterial primers 27 F and 355R [52] with Fluidigm CS1 and CS2 adapters (Illumina, San Diego, CA). PCR was conducted with the following reaction conditions: 0.1 U/µL Gotaq (Promega, Madison, WI), 1X Gotaq buffer, 0.25 mM of each dNTP, 2.5 mM MgCl_2_, and 0.25 µM of each primer, and the following thermocycler conditions: 95 °C for 5 min; 30 cycles of 95 °C for 30 s, 51 °C for 1 min, and 72 °C for 1 min; and finally 72 °C for 7 min. Amplification was confirmed on a 1% Low Electroendosmosis (LE) agarose gel. Four negative PCR controls consisting of PCR-grade water instead of DNA extract were included, confirmed to lack amplification on a gel, and sequenced alongside other samples. Libraries were prepared following Naqib et al. [53] and sequenced on three separate runs on an Illumina MiSeq platform using 250 bp paired-end reads.

Amplicon sequence data were analyzed using QIIME2 (ver2017.11) following the Moving Pictures tutorial as described by Vohsen et al. [49]. Reads were joined using vsearch [54] and quality filtered using q-score-joined. Deblur-denoise [55] was used to detect chimeras, trim assembled reads to 300 bp, and differentiate amplicon sequence variants (ASVs). Classifications of ASVs was achieved by building a classifier with the SILVA v128 SSU 99% consensus sequence database using a naïve Bayes fit through scikit-learn [56]. ASVs classified as the SUP05 cluster were used for later analyses.

### 16S rRNA gene cloning

Cloning was employed to obtain full-length 16S rRNA gene sequences of the abundant Thioglobaceae phylotypes in corals. We used the universal bacterial primers 27 F and 1492 F with the following PCR conditions on a Mastercycler pro (Eppendorf, Hamburg, Germany); 95 °C for 5 min for DNA denaturation; 30 cycles of 95 °C for 1 min, 55 °C for 1 min sec and 72 °C for 1 min; followed by a final extension at 72 °C for 10 min. PCR reactions consisted of 1X Gotaq buffer (Promega, Madison, WI), 2.5 mM MgCl_2_, 0.25 mM dNTPs, 0.4 µM of each primer, and 0.1 U/µL Gotaq (Promega) and 1 or 4 µL DNA extract in 25 µL reaction volume. PCR amplicon size was visualized on a 1% LE agarose gel (110 V for 35 min) against a size standard to confirm amplification of the correct target sequence. The PCR products were cleaned using ExoSAP-IT Express cleanup kits (Thermo Fisher, Waltham, MA) and used as templates in a TOPO TA cloning reaction (Thermo Fisher, Waltham, MA). The TOPO cloning reaction combined 4 µl of cleaned PCR product, 1 µl of salt buffer, and 1 µl of TOPO TA vector, and was incubated at room temperature for 10 min. Transformation was conducted using One Shot™ TOP10 Chemically Competent *E. coli* (Invitrogen, USA) by combining 3 µl of TOPO cloning reaction with 50 µL of *E. coli* cells. Samples were placed on ice for 30 min, heat shocked in a water bath at 42 °C for 30 s, and finally placed back on ice for 2 min. Transformed cells were mixed with 250 µL of S.O.C. medium and incubated in 37 °C for 1 h with shaking (220 rpm). After incubation, 50—150 µL of cultured cells were plated onto LB agar medium containing 50 µL X-GAL (40 mg/mL) and 50 mg kanamycin per plate and incubated for 15 h at 37 °C. Later, 8–10 white colonies were each placed in 20 µl sterilized water and 1 µl was taken as a template for final PCR amplification with either i) 27 F and 1492R primers using the same PCR conditions as above except an increased initial denaturation of 10 min or ii) M13 forward and reverse primers using an initial denaturation at 95 °C for 10 min; 32 cycles of 95 °C for 30 s, 55 °C for 30 s, and 72 °C for 45 s; and final extension at 72 °C for 10 min. Correct amplicon size was confirmed on a 1% agarose gel. PCR products were cleaned with ExoSAP-IT Express cleanup kits and sent to the Genomics Core Facility at Pennsylvania State University for Sanger sequencing with either 27F/1492R or M13 primer sets.

### Phylogenetic analyses

A phylogenetic tree was constructed to infer the evolutionary positions of the coral-associated Thioglobaceae using 16S rRNA gene sequences produced in this study through Sanger sequencing along with related sequences from publicly available databases (Table S4). Additional 16S sequences were extracted from publicly available genomes used in the phylogenomic analysis described below using barrnap ver0.9 [57]. Sequences over 1000 bp were aligned using Clustal Omega ver1.2.4 [58] and a maximum likelihood tree was constructed using IQ-TREE ver2.3.0 [59]. ModelFinder [60] was used to choose the Tamura and Nei 3 model and rate heterogeneity with four gamma categories (TPM3 + R4) based on the highest BIC score. UFBoot2 [61] was used to obtain ultrafast bootstrap support values (UFBoot) using 1000 replicates. Using this as a reference tree, Thioglobaceae ASVs (280 bp) were placed within this tree using pplacer v1.1.alpha19 [62] after realigning all sequences including ASVs with Clustal Omega. A custom script was developed to achieve this that is modified from the clustuneR package (https://github.com/PoonLab/clustuneR) and additional shell scripts.

### Stable isotope analysis

Tissue samples from twenty-two colonies of *Paramuricea* sp. B3 from AT357 were processed for stable isotopic analysis to investigate any potential trophic relationship between the coral host and their associated Thioglobaceae. These data were previously reported by Osman et al. [47]. Coral samples were collected in 2015 and 2016, frozen in liquid nitrogen upon recovery of the ROV, and stored at −80 °C for up to 12 months. Frozen tissue was dried at 47 °C for two days, pulverized, then acidified with 2–5 drops of 2 N phosphoric acid to remove calcium carbonate. Samples were then redried and acidified until all carbonate was removed as indicated by a lack of bubbling and then redried a final time. Two mg from each dried sample was encapsulated in tin and sent to UC Davis for carbon and nitrogen stable isotopic analysis. Samples were analyzed using a PDZ Europa ANCA-GSL elemental analyzer coupled to a PDZ Europa 20–20 isotope ratio mass spectrometer (Sercon, Cheshire, UK).

A linear model was constructed to test the correlation between the relative abundance of the dominant Thioglobaceae in *Paramuricea* sp. B3 and the stable isotopic composition of the coral tissue. The percentage of ASV 4 and sampling year were used as predictor variables while both δ^13^C and δ^15^N were used as response variables simultaneously.

### Metagenomes and metatranscriptomes

Metagenomes and metatranscriptomes of *Paramuricea* sp. B3 were sequenced in order to assemble genomes and assess the metabolic potential of its associated Thioglobaceae. DNA was extracted from four *Paramuricea* sp. B3 colonies using Qiagen DNA/RNA allprep kits with β-mercaptoethanol added to the RLT + lysis buffer following the manufacturer’s instructions. A NanoDrop ND-1000 Spectrophotometer was used to confirm the concentration of these DNA extracts were above 10 ng/µL. Additionally, RNA was extracted from two of those *Paramuricea* sp. B3 colonies using a Qiagen RNeasy extraction kit. The RNA extracts were enriched for bacteria by depleting coral host rRNA using a Ribo-Zero Gold Yeast kit (Illumina, San Diego, CA, USA) following the manufacturer’s protocols. RNA extracts before and after enrichment were quantified on an Agilent Bioanalyzer 2100 using a Eukaryote Total RNA Nano chip and to confirm minimal degradation. DNA extracts were sequenced on a HiSeq2500 platform using 150 bp paired-end reads to a depth of 40–50 million read pairs. The two *Paramuricea* sp. B3 Libraries with the highest coverage of Thioglobaceae were sequenced a second time for higher genomic coverage to a depth of 150 million read pairs. The RNA extracts were sequenced on a MiSeq platform using 150 bp single-end reads to a depth of 30—40 million reads.

All DNA sequence libraries were screened for Thioglobaceae SSU rRNA using phyloFlash ver3.3 [63]. The library with the highest coverage of Thioglobaceae SSU rRNA was assembled using megahit ver1.1.2 [64]. The reads from each of the four libraries were individually mapped to this assembly using bbmap with default parameters and these coverages were used to bin the assembly using metabat (ver2.12.1). A bin containing the 16S rRNA classified as Thioglobaceae and all contigs on the associated scaffolds was used as a draft genome and was annotated using RASTtk ver2.0 [65, 66]. The two RNA sequence libraries were mapped to these gene annotations using kallisto ver0.44.0 [67] to determine which genes were expressed using 100 bootstrap replicates, an estimated fragment length of 180 with a standard deviation of 20, and an index with a k-mer size of 31. The expression levels of all predicted protein-encoding genes were re-standardized by dividing the transcripts per million of each individual gene by the sum of all transcripts per million excluding rRNA genes then multiplying the resulting value by 1 000 000.

Additionally, a phylogenomic tree was constructed to compare the coral-associated Thioglobaceae genome described above to publicly available genomes. The amino acid sequences of 120 phylogenetically informative genes (bac120 gene set) [68] from each genome were extracted and aligned using the identify and align commands in the Genome Taxonomy Database and associated taxonomic toolkit (GTDB-Tk ver2.4.0) [69]. IQ-TREE ver2.3.0 was used to create a maximum Likelihood phylogenetic tree from this alignment using the Le and Gascuel 2008 model with observed amino acid frequencies and rate heterogeneity with five categories (LG + F + R5).

## Results

### Occurrence of Thioglobaceae in corals, water, and sediment

A total of 72 amplicon sequence variants (ASVs) were classified as Thioglobaceae across coral, water, and sediment samples. We considered an ASV to be abundant in a coral if it constituted > 10% of the microbial community in at least one coral sample. Fifteen ASVs were abundant in coral samples (12—91%) and were found in 13 morphotaxa from a wide depth range (~ 350—1800 m) including (ordered by depth of occurrence) *Paramuricea* sp. E, *Muriceides hirta*, *Lateothela grandiflora*, *Callistephanus* sp., *Acanthogorgia aspera*, *Callogorgia delta*, unidentified bamboo coral 1, *Chrysogorgia* sp., *Paramuricea* sp. B3, *Hemicorallium niobe*, *Callistephanus pallida*, *Paramuricea biscaya*, and *Stichopathes* sp. 3 from sites in BOEM lease blocks VK906, MC751, VK826, GC234, MC462, AT357, GB903, GC852, and MC344 (Fig. 1A,B; Table S5).

These ASVs exhibited specificity for coral hosts. Twelve were only abundant in corals within a single genus or species and were absent or rare in other corals (Fig. 1B). Further, many coral species retained the same dominant ASV across multiple sites (Ex: ASV 14 in *Chrysogorgia* sp.) while other coral species at the same sites hosted different ASVs. For example, the five co-occurring corals at site GC234 each hosted different dominant Thioglobaceae ASVs.

None of these abundant, coral-associated ASVs were ever detected in a water sample, and nine of the twelve were not detected in any sediment sample. Those present in sediment samples comprised a maximum of less than 0.2% of the sediment microbial community (Table S5). In addition, 58 other Thioglobaceae ASVs were detected that comprised less than 10% of the microbial community in individual corals. Of these, 29 were only found in coral samples and never in water or sediment samples. These ASVs were associated with *Acanthogorgia aspera, Bathypathes* sp. 1, *Callogorgia americana, Callogorgia delta, Chelidonisis aurantiaca, Trachythela rudis, Madrepora oculata, Paragorgia regalis, Paragorgia* sp. 2, *Paramuricea biscaya, Paramuricea* sp. E, *Paramuricea* sp. B3, *Stichopathes* sp. 2, *Callistephanus* sp., unidentified bamboo coral 1, and unidentified octocoral 1*.*

Other Thioglobaceae ASVs were present in water samples and together composed up to 44% of the bacterioplankton. Seven ASVs were found exclusively in water samples. Similarly, Thioglobaceae ASVs were found in sediment samples and comprised up to 55% of the microbial community. Five ASVs were exclusively found in sediment samples.

Twenty-seven nearly full-length 16S sequences (1270 bp – 1463 bp) of Thioglobaceae were obtained from five coral species through cloning: *Muriceides hirta, Acanthogorgia aspera, Callistephanus* sp., *Chrysogorgia* sp., and *Callistephanus pallida.* These sequences were most similar to the dominant Thioglobaceae ASVs in these corals differing by 0–3 bp (Table S6): ASV 1 in *M. hirta;* ASVs 8 and 10 in *A. aspera*; ASVs 6 and 7 in *Callistephanus* sp.; ASV 14 in *Chrysogorgia* sp.*;* and ASVs 1 and 15 in *Callistephanus pallida*.

### Phylogenetic positions of Thioglobaceae that associate with corals

**Fig. 3:**
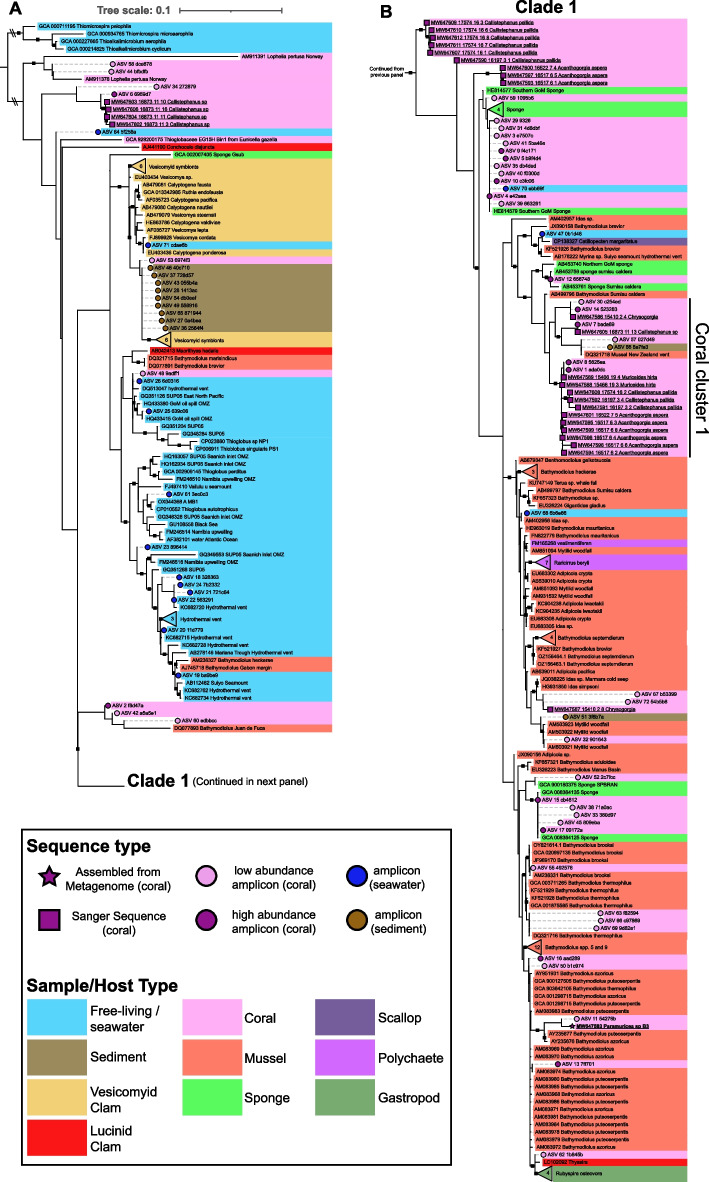
Phylogenetic tree of Thioglobaceae using the 16S rRNA gene. Maximum Likelihood phylogenetic tree of all Thioglobaceae 16S sequences generated in this study. Clade 1 (**B**) is separated from the rest of the tree in (**A**). Sequence labels are colored by sample or host type. Sequences generated in this study are denoted with symbols at tips (colored circles, squares, or a star). ASVs placed with pplacer are denoted with dashed-line branches and circles: magenta for abundant coral-associated ASVs, light pink for rare (always < 10%) coral-associated ASVs, blue if mostly found in water samples, and brown if mostly found in sediment samples. Sequences generated from cloning are underlined and denoted with magenta squares. The 16S rRNA gene extracted from the metagenome is underlined and bold, and denoted with a magenta star. Numbers inside collapsed nodes represent the number of sequences included from other studies. Nodes with greater than 95% ultrafast bootstrap support are denoted with black squares

Most coral-associated ASVs as well as longer sequences derived from cloning clustered within Clade 1 as described by Petersen et al. [20] (Fig. 3B). This clade is comprised of the chemoautotrophic symbionts of mussels, sponges, snails, and clams from hydrothermal vents, cold seeps, whale falls, and wood falls. The coral-associated members of this clade did not form a monophyletic group and were instead dispersed among several divergent lineages. However, several abundant ASVs from multiple coral species clustered near each other (Coral cluster 1, Fig. 3B). Interestingly, when corals consistently hosted two distinct Thioglobaceae ASVs (Fig. 1B: *Callistephanus* spp. and *Acanthogorgia aspera*), they belonged to divergent Lineages where one belonged to Coral Cluster 1 (Fig. 3AB). While ASV 1 was abundant in both *Muriceides hirta* and *Callistephanus pallida* (Fig. 1B), consistent sequence variation between hosts was observed in the longer sequences obtained from cloning (Fig. 3B).

Coral-associated sequences that did not cluster within Clade 1 include some sequences derived from *Callistephanus* sp., several ASVs that were low in abundance, and publicly available sequences from *Desmophyllum pertusum* and *Eunicella gazella.* Further, most ASVs detected in the water column clustered with free-living members from hydrothermal vents and oxygen minimum zones while most detected in the sediment clustered with the symbionts of vesicomyid clams (Fig. 3A).

### Occurrence of Thioglobaceae at AT357

A single Thioglobaceae ASV (ASV 4) was dominant in *Paramuricea* sp. B3 colonies at AT357 and ranged from 6–85% of the microbial community (*n* = 24, Fig. 2E). ASV 4 was also present in three of the four *Madrepora oculata* colonies and one of the three zoanthid gold coral colonies sampled at this site, but only constituted a maximum of 0.069% of their microbial communities. ASV 4 was also found in four of the ten sediment samples from this site, with a maximum relative abundance of 0.14%. This ASV was not found in any water sample from AT357, although other Thioglobaceae ASVs were present that together comprised between 2.5—5.4% of the microbial community.

### Metabolic capabilities of a coral-associated Thioglobaceae

A 1.9 Mbp draft genome of a member of the family Thioglobaceae was assembled from a *Paramuricea* sp. B3 metagenome (k-mer coverage 145). A phylogenomic tree using an amino acid alignment of the bac120 gene set placed this genome within Clade 1 near the symbionts of *Catillopecten margaritatus* from cold seeps and *Bathymodiolus septemdierum* and haplosclerid sponges from hydrothermal vents (Fig. 2F). Notably, while also within Clade 1, this is a different phylogenetic position than that predicted from the 16S rRNA gene which had lower bootstrap support (Fig. 3B). Consistent with the taxonomy proposed by Ansorge et al. [70], we propose the name *Ca. Thiomultimodus paramuricea* for this associate of *Paramuricea* sp. B3.

**Fig. 4:**
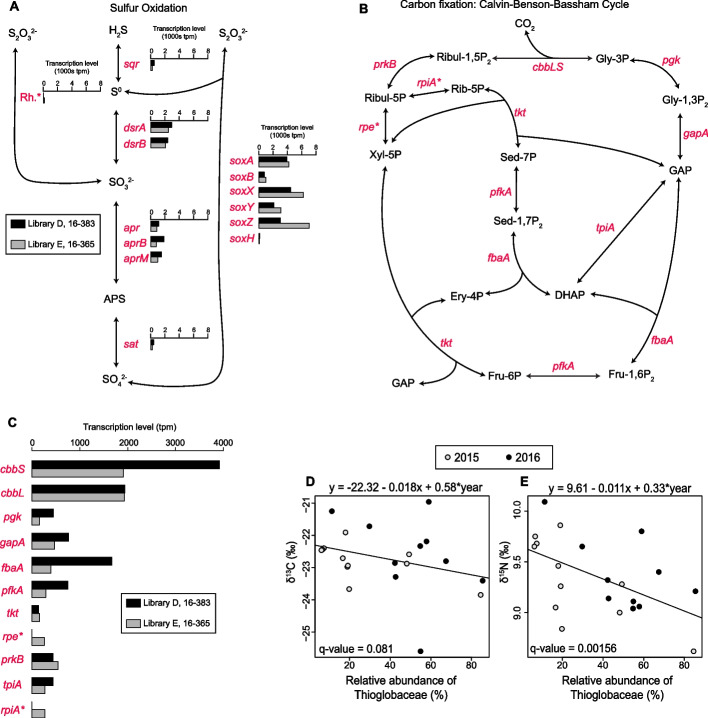
Sulfur oxidation and carbon fixation expression in *Ca. Thiomultimodus paramuricea* and stable isotope analysis. **A** Sulfur oxidation pathway and transcription levels (transcripts per million) of genes encoding enzymes involved therein. Compounds are noted in black text and genes are italized in red text. A star denotes genes with transcripts detected in only 1 RNA library. Rhodanese sulfurtransferase is abbreviated Rh. The expression levels of genes in libraries D and E are displayed in black and gray respectively. **B** Carbon fixation pathway (Calvin Benson-Bassham Cycle) and (**C**) the expression levels of genes involved. The relationship between the relative abundance of the dominant Thioglobaceae ASV in *Paramuricea* sp. B3 and the stable carbon (**D**) and nitrogen (**E**) isotopic compositions of the coral tissue at site AT357

This genome contained the gene repertoire required for sulfur-oxidation and carbon fixation. The genes necessary to oxidize the reduced sulfur species thiosulfate, elemental sulfur, and hydrogen sulfide were all present (Fig. 4A, Table S7-8). Transcripts of all of these genes were detected in both RNA libraries with the exception of rhodanese sulfurtransferase which was only detected in one library (Table S7). The genes of the sox pathway appeared to be the most highly expressed (mean transcripts per million 890–5 346) whereas sulfide quinone reductase was lower (mean tpm 356). However, the low sample size (two libraries) precludes statistical comparisons.

The genome also included the genes necessary to fix carbon through a modified Calvin-Benson-Bassham (CBB) cycle (Fig. 4B). Like other Thioglobaceae, this genome was missing the genes that encode sedoheptulose-1,7-bisphosphatase and fructose-1,6-bisphosphatase. However, the pyrophosphate-dependent 6-phosphofructokinase (*pfkA*) encoded in this genome has been suggested to perform the roles of these missing genes in chemoautotrophs [71–73]. Transcripts of all of the genes involved in the CBB cycle were detected in both RNA libraries except ribose-6-phosphate isomerase (*rpiA*) and ribulose-phosphate 3-epimerase (*rpe*) which were present in only one library each. The genes encoding the large and small chains of ribulose-bisphosphate carboxylase (RuBisCO) were the most highly expressed genes in this pathway (mean tpm 1 933 and 2 914, Fig. 4C).

Genes involved in other nutritional roles were identified in this genome. These included genes encoding assimilatory nitrate and nitrite reductases and genes involved in the biosynthetic pathways of every essential amino acid as well as vitamins such as thiamin (B1), riboflavin (B2), pyridoxine (B6), biotin (B7), and folate (B9).

### Correlation between stable isotopic composition and the relative abundance of Thioglobaceae

The stable carbon isotopic compositions of the tissue of most *Paramuricea* sp. B3 colonies were within the range of carbon fixed by surface phytoplankton (−22 to −15 ‰ δ ^13^C) [74] however four colonies were more negative with the lowest at −25.6 ‰ (Fig. 4D, Tables S9-12). The relative abundance of the dominant Thioglobaceae ASV was negatively correlated with both δ ^13^C and δ ^15^N values (p = 0.008, *n* = 21, Fig. 4D,E). However, its relative abundance was much more predictive of nitrogen (q = 0.00156, R^2^ = 0.43) than carbon (q = 0.08, R^2^ = 0.16) isotopic compositions.

## Discussion

### Thioglobaceae is a diverse and abundant family that associates with multiple deep-sea coral species primarily at seeps

A large number of distinct ASVs belonging to the family Thioglobaceae were widespread throughout the deep Gulf of Mexico and were detected in sediment, water, and multiple species of deep-sea scleractinians, antipatharians and octocorals. We propose that some members of the family Thioglobaceae form a facultatively symbiotic association with deep-sea corals sensu Goff [75]. First, individual ASVs were abundant in the microbiome of several coral species (> 10%) and most of these were not found in the surrounding sediment or water. Some coral-associated ASVs were found in the sediment at very low relative abundances which could be due to cross contamination during sample processing or errors in base calls in indexes with demultiplexing [76]. This suggests that corals are the main habitat for many Thioglobaceae and are not passive contamination from the sediment or water. Additionally, coral-associated Thioglobaceae showed host fidelity. Several abundant ASVs associated with a single species of coral, occurring in this species at multiple sites but not in co-occurring coral species (Fig. 1).

The ASVs most likely to play an important role in corals are those which occur at high relative abundance (> 10%). Such ASVs were not found in any shallow or mesophotic corals. Instead, they were primarily found in corals that grew at deep sites near signs of active seepage with the exception of two colonies collected within lease blocks MC462 and VK906 where there are no known signs of active seepage. At multiple locations, active signs of seepage were visible within 1 m of some corals. At other locations, no signs of seepage were visible in close proximity to the corals however bacterial mats or seep-associated fauna were noted in other unsampled areas of these sites.

Thioglobaceae has previously been detected in the mesophotic octocorals *Eunicella gazella* and *Alcyonium coralloides* from the Mediterranean Sea [45, 46], however no mesophotic or shallow-water corals in this study hosted Thioglobaceae at relative abundances higher than 0.29% (Table S5). Most of the abundant coral-associated members of Thioglobaceae from this study including *Ca. Thiomultimodus paramuricea* clustered within Clade 1, however they did not form a monophyletic group but rather belonged to multiple divergent lineages. Interestingly, the associate of *Eunicella* fell outside of Clade 1 and was unrelated to any of the Thioglobaceae sequences obtained from corals in this study (Figs. 2, 3). Also, that associate’s genome lacked sulfur oxidation genes present in the genome of *Ca. Thiomultimodus paramuricea* but did encode dissimilatory sulfate reductase and RuBisCo [45]. Similarly, the previously reported associate of *Desmophyllum pertusum* fell outside Clade 1 along with two ASVs only found at low abundance in corals (Fig. 3: ASVs 44, 58; 0.96% max abundance). Therefore, Thioglobaceae may be widespread coral associates but each Lineage may differ in metabolic capabilities where only certain Lineages are associated with cold seeps such as those within Clade 1. Further, some divergent lineages co-occurred within the same hosts suggesting potential metabolic complementarity between these lineages as occurs in some *Bathymodiolus* symbionts [77].

In addition to comprising multiple lineages within Thioglobaceae, their coral hosts belonged to multiple families and orders suggesting that this association evolved multiple times. This is similar to the phylogeny of the symbionts of Bathymodiolin mussels which do not form a monophyletic group [20] and whose hosts are thought to have invaded seeps and hydrothermal vents multiple times throughout their evolutionary history [22, 78, 79].

The phylogenetic position of *Paramuricea* sp. B3’s associate was not consistent when inferred from the 16S rRNA gene compared to the bac120 gene set. This may be due to horizontal gene transfer which is known to be widespread in this family [28, 70]. Obtaining genomes of other coral-associated Thioglobaceae should help to unravel their evolutionary histories and any differences in metabolic capabilities.

### The metabolic capabilities of coral-associated Thioglobaceae

We hypothesize that some coral-associated Thioglobaceae, including *Ca. Thiomultimodus paramuricea,* supplement their coral hosts near seeps with chemoautrophic primary productivity through the oxidation of reduced sulfur compounds and carbon fixation via the Calvin-Benson-Bassham Cycle.

It was previously proposed that cnidarians could not associate with sulfur-oxidizing symbionts due to the high demand for oxygen [44]. However this was largely based on the symbionts of tubeworms which belong to unrelated gammaproteobacteria [80] whereas Thioglobaceae are now thought to have the highest affinity sulfur uptake systems compared to any other organism and any other substrate [81]. They can oxidize sulfide at concentrations as low as 5 nM which is below the detection limit of common measurement methods, and can oxidize nearly all available sulfide in their environment masking active sulfur cycling [81]. Further, several studies suggest that the SUP05 clade can also oxidize sulfide under hypoxic or anoxic conditions by using nitrate as an oxidizer [82–85]. In anoxic conditions, the respiratory nitrate reductase of the sulfur-oxidizing epsilonproteobacterium, *Sulfurimonas gotlandica,* strongly fractionates the remaining nitrate after denitrification [86]. If the assimilatory nitrate reductase encoded in the genome of *Ca. Thiomultimodus paramuricea* is similarly coupled to sulfur oxidation in low oxygen conditions*,* then it is possible that the nitrogen isotope compositions of *Paramuricea* sp. B3 may reflect assimilation of the resulting ammonia.

Alternatively, coral-associated Thioglobaceae may primarily oxidize thiosulfate as opposed to hydrogen sulfide. Chemosymbiotic animals that utilize hydrogen sulfide are often embedded in the sediment and growing closer to the source of sulfide whereas corals are upright in the water column where sulfide concentrations are lower due to its high reactivity with oxygen [44]. Thiosulfate has a longer residence time and may exist at high enough concentrations around corals to support their Thioglobaceae. This may be reflected in the apparently higher transcription levels of the *sox* genes which oxidize thiosulfate compared to *sqr* which oxidizes hydrogen sulfide. To clarify this, careful chemical characterization of these coral habitats is needed that measures multiple sulfur species in close proximity to coral colonies.

### Interaction with the coral host and its consequences

In *Paramuricea* sp. B3, we found that the relative abundance of *Ca. Thiomultimodus paramuricea* (ASV 4) was negatively correlated with the stable isotopic composition of the coral tissue suggesting that the holobiont receives some contribution from chemoautotrophy. The mechanism of supplementation is still uncertain, however a possibility is that corals occasionally digest a percentage of their symbiont population as in *Bathymodiolus* spp. [87]. Alternatively, there may be selective transfer of a limited number of organic compounds from the symbiont to host. However, the stable isotope analysis suggests this nutritional input is quite limited. Still, this may be very important to the coral host since deep-sea corals are very long-lived (> 500 years for some *Paramuricea* spp.) and are food-limited [88–90].

Although our results strongly suggest a relationship between Thioglobaceae and coral hosts, it does not demonstrate an unequivocal nutritional link. For example, the isotopic signal may simply arise from the bacteria themselves. Alternatively, the stable isotope data may reflect nutritional input from other chemoautotrophically-based food sources such as resuspended bacteria from mats or small invertebrates that graze on mats.

Alternatively, this symbiont could also be providing the coral with essential amino acids or vitamins which may be lacking in marine snow. Genes necessary to synthesize all essential amino acids were present as were genes to synthesize five vitamins (B1,2,6,7,9). Finally, Thioglobaceae may simply serve to detoxify hydrogen sulfide in coral tissue by oxidizing it into elemental sulfur.

Understanding where Thioglobaceae reside within corals would shed light on their interactions however this remains unknown. It seems unlikely that Thioglobaceae inhabit the mucus since most of the abundant ASVs were not detected in water samples, octocorals only have a thin layer of mucus, and they were rinsed before preservation. They may reside within the tentacle epidermis as they do in anemones [29]. Microscopy is required to confirm the location of these symbionts in corals.

## Conclusion

This work has revealed a previously unknown and unexpected association between deep-sea corals at seeps and bacteria from the widespread and ecologically important family Thioglobaceae. These bacteria exist throughout the world’s oceans where they strongly influence biogeochemical cycles and associate with foundation species at hydrothermal vents and cold seeps. We provide the first indications that this group may be widespread symbionts of deep-sea corals at seeps which support distinct and diverse animal communities in the deep sea. This may represent an important mechanism of ecosystem function in coral communities where they overlap with seeps by supplying nutrition to these food-limited habitats. Our findings suggest that the footprint of chemosynthetic environments is wider than currently understood.

## Supplementary Information

Additional File 1: Supplementary Tables S1-S12. Includes (S1) sample breakdown by site, (S2) sample metadata, (S3) McLane pump metadata, (S4) accessions of sequences used in trees, (S5) ASV table, (S6) blastn results of ASVs against cloned sequences, (S7) RASTtk genome annotations and kallisto expression levels, (S8) Classic RAST annotations (S9-12) stable isotope data and statistical analyses.

## Data Availability

Raw sequence data for metagenomes, the 16S survey, and metatransciptomes as well as the genome assembly for Ca. *Thiomultimodus paramuricea* were deposited in National Center for Biotechnology Information (NCBI) Sequence Read Archive (SRA) under BioProject numbers PRJNA565265 and PRJNA574146. Full-length 16S rRNA sequences generated in this study are available under accessions MW647583, MW647586-612. Data are also publicly available through the Gulf of Mexico Research Initiative Information & Data Cooperative (GRIIDC) at (https://griidc.org) (doi: < https://doi.org/10.7266/RYMQTDQ9 >, R4.x268.000:0125, and R4.x268.000:0125). Code used for bioinformatics analyses is available on Figshare at https://doi.org/10.6084/m9.figshare.28453541.
